# Cytotoxicity, Mitochondrial Functionality, and Redox Status of Human Conjunctival Cells after Short and Chronic Exposure to Preservative-Free Bimatoprost 0.03% and 0.01%: An In Vitro Comparative Study

**DOI:** 10.3390/ijms232214113

**Published:** 2022-11-15

**Authors:** Sabrina Petricca, Giuseppe Celenza, Ciro Costagliola, Fausto Tranfa, Roberto Iorio

**Affiliations:** 1 Department of Biotechnological and Applied Clinical Sciences, University of L’Aquila, 67100 L’Aquila, Italy; 2 Department of Neurosciences, Reproductive and Dentistry Sciences, University of Federico II, 80131 Naples, Italy

**Keywords:** bimatoprost, preservative-free, glaucoma, human conjunctival epithelial cells, cell cycle, mitochondrial activity, ROS, apoptosis

## Abstract

Prostaglandin analogues (PGAs), including bimatoprost (BIM), are generally the first-line therapy for glaucoma due to their greater efficacy, safety, and convenience of use. Commercial solutions of preservative-free BIM (BIM 0.03% and 0.01%) are already available, although their topical application may result in ocular discomfort. This study aimed to evaluate the in vitro effects of preservative-free BIM 0.03% vs. 0.01% in the human conjunctival epithelial (HCE) cell line. Our results showed that long-term exposure to BIM 0.03% ensues a significant decrease in cell proliferation and viability. Furthermore, these events were associated with cell cycle arrest, apoptosis, and alterations of ΔΨ_m_. BIM 0.01% does not exhibit cytotoxicity, and no negative influence on conjunctival cell growth and viability or mitochondrial activity has been observed. Short-time exposure also demonstrates the ability of BIM 0.03% to trigger reactive oxygen species (ROS) production and mitochondrial hyperpolarisation. An in silico drug network interaction was also performed to explore known and predicted interactions of BIM with proteins potentially involved in mitochondrial membrane potential dissipation. Our findings overall strongly reveal better cellular tolerability of BIM 0.01% vs. BIM 0.03% in HCE cells.

## 1. Introduction

Glaucoma is often referred to as “the silent thief of sight” because it usually occurs with no symptoms in most cases. It remains the second most recurrent cause of irreversible blindness globally, affecting about 76 million people [1]. Furthermore, it has been estimated that this number will increase to 112 million in 2040. Vision loss is classically associated with axonal degeneration and retinal ganglion cells (RGCs) death. In most cases, as the direct consequence of ocular hypertension [2]. Indeed, elevated intraocular pressure (IOP) is the primary and only modifiable risk factor for glaucoma. Therefore, current therapeutic strategies are addressed to lower IOP by suppressing aqueous humour inflow and/or facilitating its outflow. Medical glaucoma treatment and management options may include laser therapy, oral medications, and eye drops prescription, depending on the patient’s clinical situation. In this respect, medical therapy remains the treatment of choice for many patients, especially in the case of open-angle glaucoma. Different drugs and formulations are currently available in Europe, including prostaglandin analogues (PGAs), carbonic anhydrase inhibitors, beta-blockers, Rho-kinase inhibitors, miotics, and sympathomimetics [1].

In most cases, effective management of IOP requires long-term topical treatments with the advantage of having no marked systemic effects. However, long-term use of topical anti-glaucoma drugs may cause local side effects, such as allergy, conjunctival hyperemia [3,4,5], functional and morphological alterations of the cornea [6], eye surface inflammation, dry eye syndrome, failure of filtration surgery [7,8,9,10], as well as meibomian gland dysfunction [11,12]. In the context of self-administered therapies, patient adherence to therapy is crucial for its effectiveness. Therefore, low-frequency dosing and reduced side effects could improve patient compliance.

PGAs are often selected as first-line therapy for their greater efficacy, safety, and convenience of use (once-daily dosing) [13,14,15,16,17]. Bimatoprost (BIM), a prostaglandin F2-alpha (PGF-2 alpha) analogue, effectively reduces and controls circadian IOP. It acts primarily by remodelling the extracellular matrix of the ciliary muscle, increasing the trabecular and uveoscleral outflow of aqueous humour [18]. Unlike F prostanoid (FP) receptor agonists latanoprost and travoprost, BIM may bind and activate prostamide receptors [19], though this issue is still controversial and requires deeper investigations. BIM 0.03% has a superior IOP-lowering effect than other PGA monotherapies and a favourable tolerability profile over long-term use [14,15,20,21]. In clinical studies, its efficacy and tolerability have also been evidenced in patients with normal-tension glaucoma [22,23,24,25,26].

Nevertheless, patients treated with BIM 0.03% experience adverse events, frequently conjunctival hyperemia, increasing the chances of non-adherence to therapy [7,27,28,29]. These observations led to the development of a formulation of BIM 0.01% containing 0.02% benzalkonium chloride (BAC), which was expected to improve the drug tolerability and corneal penetration [30], while maintaining the same efficacy as BIM 0.03% in lowering IOP [31].

The BAC containing BIM 0.01% formulation reduced the frequency and severity of conjunctival hyperemia and increased goblet cell density, compared to BIM 0.03%, in patients with elevated IOP due to open-angle glaucoma or ocular hypertension [31,32].

However, preservatives in commercial formulations may influence BIM tolerance, as they may play a primary role in the ocular surface toxicity of eye drops, which may reasonably extend to the deeper compartments of the eye. Indeed, several experimental and clinical studies have demonstrated that preservatives in multidose eye drops, including BAC, are the main factors in developing ocular surface disease (OSD). BAC can exert cytotoxic effects on conjunctival and corneal epithelial cells, trabecular meshwork, and ciliary epithelial cells [33,34]. In a rat model, it has been demonstrated that BAC can also reach deeper tissues, such as the posterior chamber and the optic nerve [35,36]. In addition, it can promote mitochondrial oxidative stress and fragmentation [37,38]. In this regard, it must be considered that the RGC is a neuronal cell type highly vulnerable to mitochondrial dysfunction to the extent that it exhibits an altered inflammatory response in glaucomatous patients. [39,40]. Therefore, BAC is active in inducing and/or exacerbating OSD in glaucomatous patients [41]. It can harm patients under chronic therapy leading to severe morbidity, non-adherence and -persistence with drug treatment, and unfavourable surgical outcomes. In this regard, the European Medicine Agency (EMA) and the European Glaucoma Society (EGS) have provided specific indications to avoid preservatives in eye drops [42]. In particular, drugs without BAC should be used in glaucomatous patients with pre-existing OSD or those susceptible to dry eyes. Fortunately, preservative-free topical formulations for glaucoma treatment, some of which exhibit less toxicity than their corresponding formulations with BAC, are already available: e.g., 0.05% latanoprost, 0.1% timolol, 0.0015% tafluprost, 0.15% brimonidine, 0.03% and 0.01% BIM [33]. However, since the total absence of adverse effects of the active ingredients cannot be avoided, determining the possible adverse effects of BIM preservative-free as early as possible is also essential in the perspective of long-term therapies.

In a recent study by Medina et al. [29], a cohort of 40 patients underwent a switch from BIM 0.01% with BAC (23 patients) and preservative-free BIM 0.03% (17 patients) to a BIM 0.01% BAC-free formulation. The study has demonstrated that preservative-free BIM 0.01% has a better tolerability profile associated with non-therapeutical inferiority in the control of IOP compared to the other BIM formulations.

So far, no comparative study of cytotoxicity between 0.03% and 0.01% BIM preservative-free has been conducted. Given the above, this study aims to investigate the cellular basis of the better tolerability of BAC-free BIM 0.01% to BIM 0.03% in the human conjunctival epithelial cell line (Wong–Kilbourne derivative of Chang conjunctiva). To this end, cell proliferation, viability, and cell cycle profiles were analysed. In addition, reactive oxygen species (ROS) production was assayed. Given mitochondria’s critical role in ROS generation and cell death, the ability of BIM to alter mitochondrial membrane potential (ΔΨ_m_) and the occurrence of apoptosis were also examined.

An in silico drug network interaction was also performed to explore known and predicted interactions of bimatoprost with proteins potentially involved in the mitochondrial membrane potential dissipation.

## 2. Results

### 2.1. Cell Growth and Viability of HCE Cells Are Not Affected by BIM 0.01%

**Figure 1:**
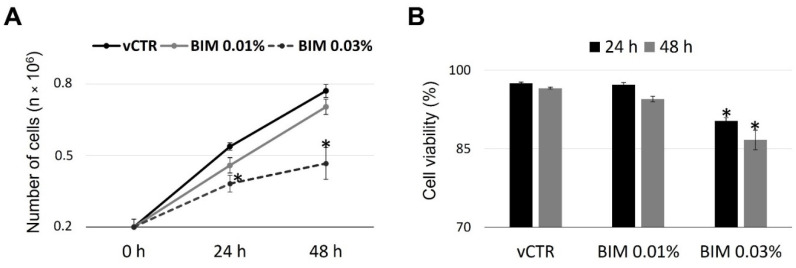
Bimatoprost (BIM) 0.01% does not induce changes in human conjunctival epithelial (HCE) cells proliferation and viability. Cell growth (**A**) and viability (**B**) rate in control (vCTR) and treated HCE cells incubated for 24 and 48 h with BIM 0.01% and 0.03%. BIM 0.03% induces a significant decrease in cell growth and viability at 24 and 48 h. Results are the media of three independent experiments ± SE; one-way ANOVA (Dunnett’s method); * *p* < 0.05 compared with vCTR.

Early experiments on HCE cells, incubated with BIM 0.03% or BIM 0.01% for 24 h and 48 h, revealed differential effects on cell growth (Figure 1A) and viability (Figure 1B) evaluated by Trypan-blue exclusion assay. Although a significant reduction in the proliferation rate or viability was found when comparing BIM 0.03% treated samples with vCTR group, no substantial changes were reported for BIM 0.01% treatment. In addition, short-term exposure (30 min and 2 h) to BIM 0.03% did not induce any variations in cell proliferation and viability.

### 2.2. BIM 0.03% Affects Cell Cycle Phase Distribution, Inducing S/G2 Transition Arrest

**Figure 2:**
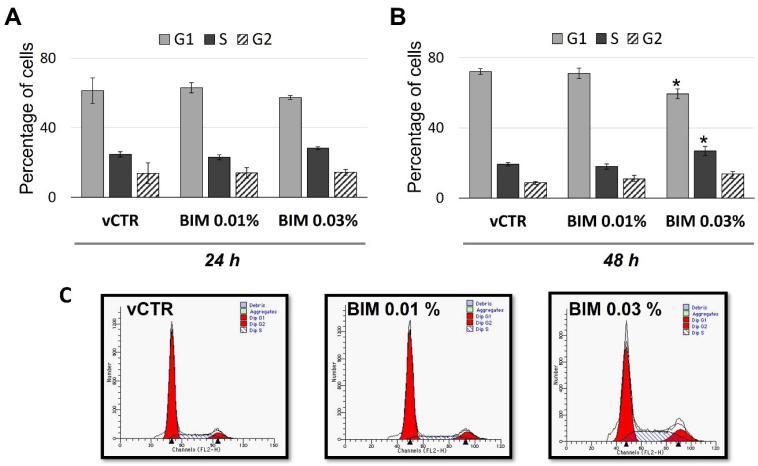
BIM 0.03% induces S/G2 arrest in HCE cells. Long-term exposure analyses of cell cycle phases distribution (percentage of cells) in HCE cells incubated in the absence (vCTR) or the presence of BIM 0.01% and 0.03% for 24 (**A**) and 48 h (**B**); BIM 0.03% induces a significant increment in the percentage of cells in S phase, followed by a slight increase in G2 phase, at 48 h of exposure; on the contrary, no effects for BIM 0.01% are detected. (**C**) Representative FACS profiles of cell cycle phases analyses at 48 h treatment. Values from three independent experiments are expressed as the media ± SE; one-way ANOVA (Dunnett’s method); * *p* < 0.05 compared with vCTR.

To further characterise the BIM 0.03%-induced cell growth inhibition, flow cytometry analyses of the distribution of cell cycle phases were performed. Our results at 24 h (Figure 2A) and 48 h (Figure 2B,C) of exposure did not show substantial differences between vCTR and BIM 0.01% treated groups, while significant differences were detected in BIM 0.03% treated groups at 48 h of exposure; in particular, we observed a significant increase of cells in S phase, about 39%, accompanied by a slight increase in G2 phase, revealing a BIM 0.03%-induced S/G2 arrest. Notably, a significant decrease in the G1 phase, nearly 18%, was also detected compared to the vCTR group.

### 2.3. BIM 0.03% Induces Biphasic Modulation in Mitochondrial Activity and a Short-Term Increase of Intracellular ROS in HCE Cells

**Figure 3:**
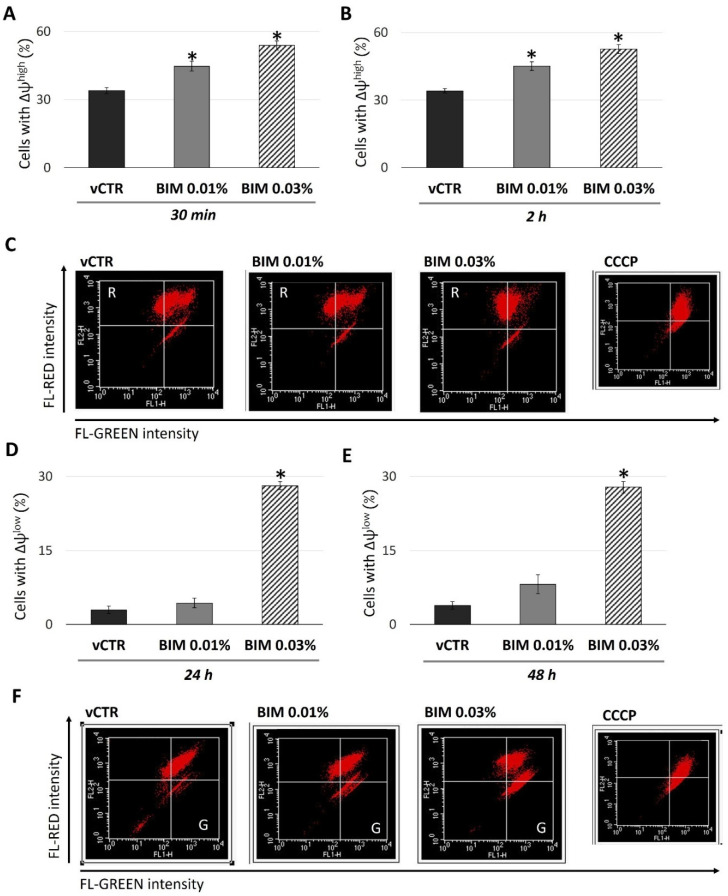
Time-dependent biphasic pattern of the mitochondrial functional activity induced by BIM 0.03%. Analyses of ΔΨ_m_ in vCTR and treated cells with BIM 0.01% and 0.03% for 30 min (**A**) and 2 h (**B**). (**C**) Representative FACS images of ΔΨ_m_, analysed by JC-1 probe, at 2 h of incubation. We used 8 µM carbonyl-cyanide-3-chlorophenylhydrazone (CCCP) for 1 h at standard conditions as a positive control for the abolishment of ΔΨ_m_. A significant increase of cells with a high ΔΨ_m_ (R, red fluorescence quadrant) is revealed at 30 min and 2 h of exposure to BIM 0.01% and 0.03%. Analyses of ΔΨ_m_ in vCTR and treated cells with BIM 0.01% and 0.03% after long-term exposure, 24 (**D**) and 48 h (**E**). (**F**) Representative FACS images of ΔΨ_m_, analysed by JC-1 probe, at 48 h of incubation. BIM 0.03% induces a significant increase of cells with a low ΔΨ_m_ (G, green fluorescence quadrant), while no significant depolarising event is detected in BIM 0.01% treated cells. Data from three independent experiments are expressed as the media ± SE; one-way ANOVA (Dunnett’s method); * *p* < 0.05 compared with vCTR.

We next examined the redox status and mitochondrial activity in HCE cells to investigate the underlying cellular events coupled with changes in proliferation and viability. To this end, we analysed the mitochondrial functional state during the time; in particular, we examined the variations in the ∆Ψ_m_ by JC-1 staining induced by BIM 0.03% and BIM 0.01% in short exposure (30 min and 2 h) and long-term exposure (24 h and 48 h). As the early effect, our results (Figure 3A–C) showed an increase of cells with ∆Ψ_m_^high^ in BIM 0.03% (about 55% of cells with ∆Ψ_m_^high^ versus vCTR); however, although to a lesser extent, a mild effect of BIM 0.01% was also detected (about 21% of cells with ∆Ψ_m_^high^ versus vCTR).

**Figure 4:**
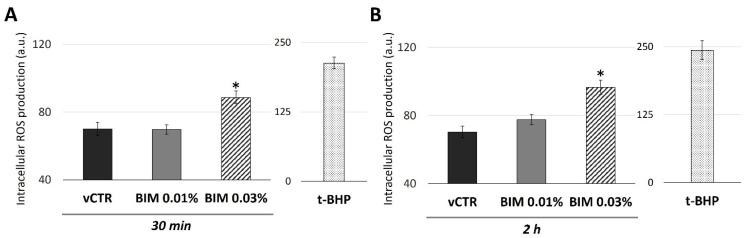
BIM 0.03% induces mild production of intracellular reactive oxygen species (ROS) in HCE cells. ROS production in HCE cells incubated in the absence (vCTR) or the presence of BIM 0.01% and BIM 0.03% for 30 min (**A**) and 2 h (**B**); intracellular ROS production was revealed by fluorimeter, detecting DCFH-DA emission in terms of fluorescence intensity (arbitrary units, a.u.). Cells treated with 200 µM tert-butyl hydroperoxide (t-BHP) were used as a positive control. Values from three independent experiments are expressed as the media ± SE; one-way ANOVA (Dunnett’s method); * *p* < 0.05 compared with vCTR.

Given that perturbations in the mitochondrial activity are well known to influence ROS production, we investigated this possibility. HCE cells were incubated with BIM 0.03% and BIM 0.01% for 30 min and 2 h, and then analysed for DCFH-DA fluorescence. Our results revealed that BIM 0.03%-induced changes in mitochondrial activity were associated with a mild ROS generation (30 min and 2 h of exposure) (Figure 4). This may suggest a global altered cellular redox status attributable to BIM 0.03% exposure, while no substantial changes were reported for BIM 0.01% treatment.

In addition, a normal level of the cellular pool with depolarised mitochondria (∆Ψ_m_^low^) was detected in BIM 0.01% groups up to 48 h of exposure; by contrast, a significant and marked drop in ∆Ψ_m_ was observed in BIM 0.03% exposed groups at 24 (Figure 3D) and 48 h (Figure 3E,F), expressing a percentage of cells with ∆Ψ_m_^low^ near to 30% versus vCTR.

### 2.4. BIM 0.03% Promotes HCE Cell Death as Both Apoptosis and Necrosis

**Figure 5:**
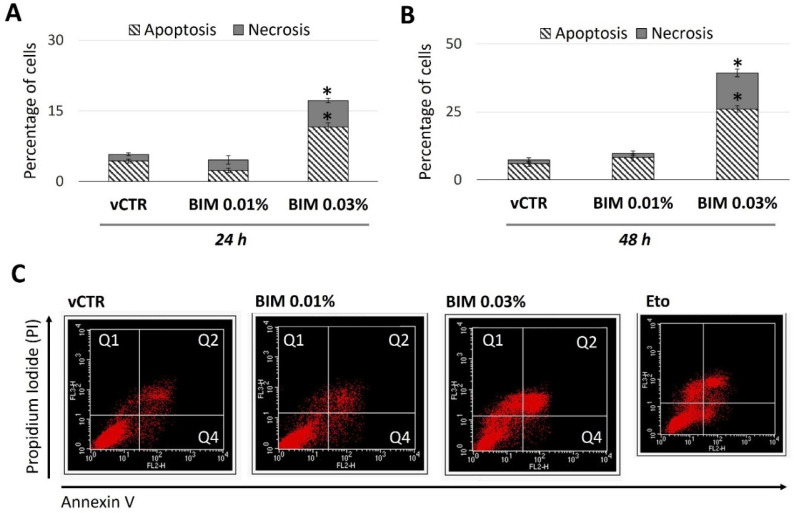
Increased levels of apoptosis and necrosis in BIM 0.03%-exposed cells. Cell death analyses (percentage of cells) in HCE cells incubated in the absence (vCTR) or the presence of BIM 0.01% and 0.03% for 24 (**A**) and 48 h (**B**); BIM 0.03% induces significant changes in the percentage of cells undergoing apoptosis and necrosis, at 24 and 48 h; no relevant effects for BIM 0.01% are detected. (**C**) Representative FACS images of AnnexinV/PI analyses, at 48 h treatment (Q1 = necrosis; Q2 = late apoptosis; Q4 = early apoptosis); cells treated with 25 µM Etoposide (Eto) for 24 h were used as a positive control for apoptosis induction. Values from three independent experiments are expressed as the media ± SE; one-way ANOVA (Dunnett’s method); * *p* < 0.05 compared with vCTR.

A decrease in ∆Ψ_m_ is often associated with increased cell death events by apoptosis or necrosis. Flow cytometry analyses were performed after Annexin V/PI double staining to characterise the BIM-induced cytotoxicity further. At 24 h (Figure 5A) and 48 h (Figure 5B,C) of exposure, our results did not show substantial differences between the control (vCTR) and BIM 0.01% treated groups. On the contrary, compared with the vCTR group, the percentage of BIM 0.03%-treated cells undergoing early (Q4) and late (Q2) apoptosis significantly increased at 24 h and 48 h.

### 2.5. Drug Network Prediction

The STITCH database was used to explore and define the plausible network of chemical and protein relations between bimatoprost and the observed mitochondrial membrane potential dissipation by including PGE_2_, bimatoprost, the malate-aspartate shuttle and the mitochondrial uncoupling proteins UCPs.

**Figure 6:**
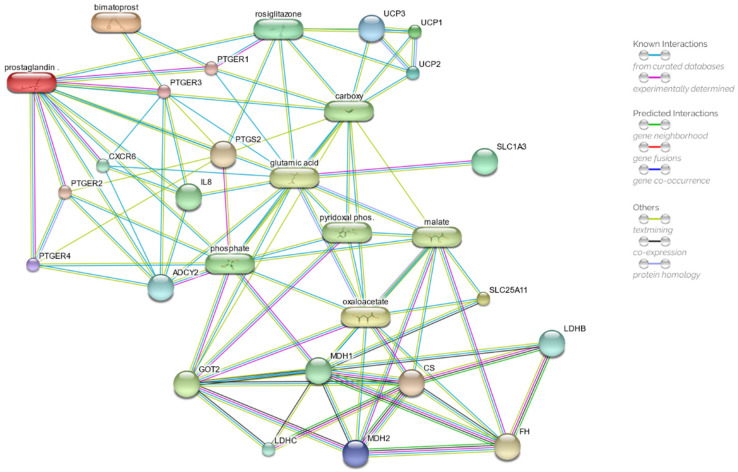
Drug–target network, obtained by STITCH database, of prostaglandin E2 (PGE_2_) and bimatoprost. Chemicals are represented as pills; proteins are represented as spheres. ADCY2, adenylate cyclase 2; CS, citrate synthase; CXCR6, chemokine (C-X-C motif) receptor 6; FH, fumarate hydratase; GOT2, mitochondrial glutamic-oxaloacetic transaminases 2; IL8, interleukin 8; LDHB, lactate dehydrogenase B; LDHC, lactate dehydrogenase C; MDHs, malate dehydrogenases; PTGERs, prostaglandin E receptors; SLC1A3, solute carrier family 1, member 3; SLC25A11, solute carrier family 25, member 11; UCPs, uncoupling proteins. More detailed information and the interactive network can be retrieved at the link: http://stitch.embl.de/cgi/network.pl?taskId=XymGQyDgGV6u (accessed on 11 November 2022).

The results of the first round of analysis, depicted in Figure 6, showed a PPI (protein–protein interaction) enrichment *p*-value equal to 7.0 × 10^−4^ and a clustering coefficient of 0.77, which strongly suggests more significant interactions among the network proteins than expected for a random set of proteins. As shown in Table S2, the lowest combined score has been estimated at 0.983 for the adenylate cyclase 2 (ASCY2, UniProt Q08462), and the highest value for the prostaglandin E receptor 4 (PTGER4, UniProt P35408) with a value of 0.999. The recorded mean combined score value is 0.991.

The predicted interaction network showed a direct relation of bimatoprost with the prostaglandin E receptors 1 and 3 (PTGER1 and PTGER3) with a combined score of 0.827 and 0.824, respectively. Combined scores higher than 0.900 can be observed between the prostaglandin receptors PTGER1 and PTGER3 with the malate-aspartate shuttle through the glutamic acid node and the fatty acid metabolism (carboxy node), which is, in turn, connected with the mitochondrial uncoupling proteins (UCP1, UCP2 and UCP3) with combined scores of 0.990.

It is intriguing that the appearance of rosiglitazone and its direct relation with the PTGER1 and the prostaglandin E2 (prostaglandin node), are characterised by combined scores higher than 0.9, precisely 0.920 and 0.929, respectively.

**Figure 7:**
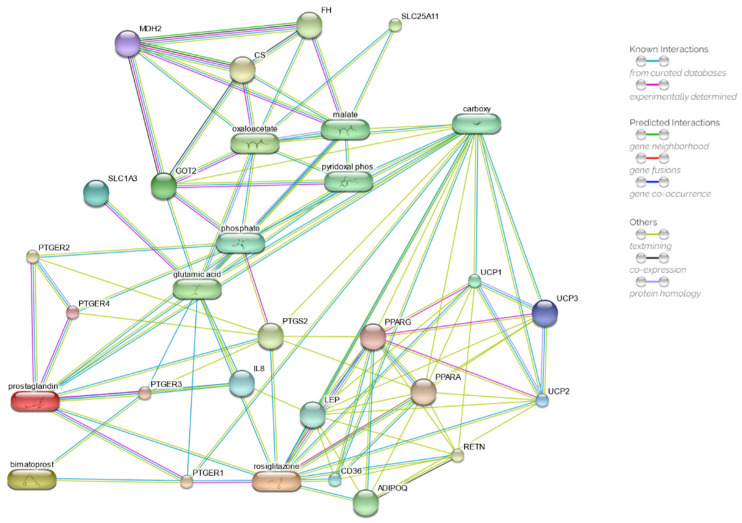
Drug–target network, obtained by STITCH database, of prostaglandin E2 (PGE2), bimatoprost, and rosiglitazone. Chemicals are represented as pills; proteins are represented as spheres. ADIPOQ, adiponectin; CD36, thrombospondin receptor; CS, citrate synthase; FH, fumarate hydratase; GOT2, mitochondrial glutamic-oxaloacetic transaminases 2; IL8, interleukin 8; LEP, leptin; MDH2, mitochondrial malate dehydrogenase 2; PPARA, peroxisome proliferator-activated receptor alpha; PPARG, peroxisome proliferator-activated receptor gamma; PTGERs, prostaglandin E receptors; PTGS2, prostaglandin E receptor 2 (subtype EP2); RETN, resistin; SLC1A3, solute carrier family 1, member 3; SLC25A11, solute carrier family 25, member 11; UCPs, uncoupling proteins. More detailed information and the interactive network can be retrieved at the link: http://stitch.embl.de/cgi/network.pl?taskId=ncdxT1FhAA9i (accessed on 11 November 2022).

A second network was then constructed based on the initial model by adding rosiglitazone to the input molecules. The second network, depicted in Figure 7, returns a PPI enrichment *p*-value of 6.38 × 10^−5^ and a clustering coefficient of 0.747. As shown in Table S3, the lowest combined score has been estimated at 0.988 for the interleukin 8 (UniProt P10145), with an overall mean value of 0.994.

In addition to the already predicted direct interaction between PGE_2_ and rosiglitazone, as well as bimatoprost and rosiglitazone through PTGER1, it was possible to observe in the predicted network the appearance of the peroxisome proliferator-activated receptors (PPARs). The highest combined scores recorded were between rosiglitazone and its known target PPARγ (0.999) and the last one with the UCP proteins: 0.949, 0.946, and 0.942 for UCP1, UCP2, and UCP3, respectively.

## 3. Discussion

As far as our knowledge, this is the first study comparing the potential of preservative-free 0.01% and 0.03% BIM in inducing cytotoxicity in a human conjunctival cell model. Our results revealed that BIM-induced effects are concentration- and time-dependent. In particular, long-term exposure (24 and 48 h) to BIM at 0.03% significantly reduces cell proliferation and viability. Furthermore, these events were associated with cell cycle arrest, apoptosis, and mitochondrial activity alterations. BIM 0.01%, on the other hand, has no cytotoxicity, no negative influence on conjunctival cell growth and viability, or mitochondrial activity. Short-time exposure (30 min and 2 h) also reveals the ability of BIM 0.03% to promote ROS generation and mitochondrial hyperpolarisation. However, positive changes in ΔΨm, though to a lesser extent, are also found in cells exposed to BIM 0.01%.

Concerning the exposition times, the short periods could reproduce the pharmacokinetics of BIM. After topical ocular dosing, BIM penetrates the human cornea and sclera, reaching the peak plasma concentration in 10 min. In addition, its elimination half-life is about 45 min (FDA “new drug therapy approvals 2017”) [43]. Moreover, to better characterise this compound, analyses were also carried out after a more extended period of time in order to simulate a situation that may approach clinical scenarios. In this sense, patients with primary glaucoma can receive medical treatment for most of their lives. Although our in vitro experiments cannot accurately predict in vivo adverse effects due to the complex structure of the ocular surface, our results support the study by Medina et al. [29], demonstrating the better tolerability of preservative-free BIM 0.01% compared to preservative-free BIM 0.03% administration, in a cohort study of 40 patients.

The mitochondrial activity and redox state play a crucial and essential role in cellular homeostasis [44,45] and many aspects of ocular health, including OSD development [46,47]. On the other hand, it has been reported that 35% of pharmaceutically relevant compounds can induce mitochondrial toxicity [48,49]. Our data clearly indicates that BIM 0.03% can affect mitochondrial biology by lowering ΔΨ_m_. The ΔΨ_m_ is an index of the function and morphology of mitochondria, as well as their energetic and redox state [50,51,52,53,54]. It plays a crucial part in ROS generation, mitochondrial homeostasis, and quality control through the selective elimination of damaged mitochondria [44,55]. The mitochondrial network (MN) constantly satisfies the cellular energy requirements to ensure proper cell cycle progression [56]. During the S/G2 transition, MN becomes more organised and interconnected as cells require more energy. In turn, mitochondrial fusion machinery needs a certain ΔΨ_m_. Thus, cellular-stress-induced ΔΨm decline can stop cell cycle progression [57]. Consistent with this, BIM 0.03%-induced mitochondrial depolarisation may arrest cells in the S phase. Our findings also support a previous study showing that Prostaglandin E2-induced ΔΨ_m_ decline regulates the expression of a set of cell-cycle-related genes in macrophages [58].

Although ΔΨ_m_ loss is usually considered as intimately related to the apoptotic process [59], the first effective response to stress is the increase in ΔΨ_m_. In this regard, many studies reported increased ΔΨ_m_ in various cell types [60,61,62,63]. Therefore, dissipation of ΔΨ_m_ is preceded by mitochondrial hyperpolarisation and generation of ROS, which thus constitutes early apoptotic events [64,65]. In line with this, short-time exposure to BIM may trigger early events of cellular cytotoxicity. However, only BIM 0.03% induced a significant increase in ROS production, as well as higher levels of ΔΨ_m_^high^ than BIM 0.01%, as described above. Therefore, the different magnitude of BIM-induced stress may have a crucial role in the decision of conjunctival cells to undergo apoptosis instead of activating adaptive responses.

The drug–protein prediction network was performed to delineate the hypothetical relationship between bimatoprost exposure and the observed mitochondrial membrane depolarisation. The in silico analysis predicted a direct relation between bimatoprost and the EP1 receptor subtype. Although it is known that PTGERs bind PGE_2_ preferentially [66], with a binding affinity for the EP_1_ subtype receptor, calculated as a constant of inhibition *K*_i_, equal to 26 nM [67], the binding affinity of bimatoprost for the EP_1_ subtype is comparable to the binding affinity for the prostaglandin receptor FP, 95 nM versus 83 nM, respectively [67]. Hence, we can easily suppose that bimatoprost can simulate the binding of PGE_2_ to PTGER1 in activating the same cascade of events observed after exposure to PGE_2_. It is not by chance that bimatoprost seems to behave as PGE_2_ in altering the mitochondrial membrane potential. For instance, PGE_2_ has been demonstrated to cause the decline of the mitochondrial membrane potential in IL-4-activated macrophages due to the altered expression of many malate-aspartate shuttle genes, specifically *Got1* [58]. The key events of apoptosis, including mitochondrial membrane potential dissipation, were observed in K562 human leukaemia cells exposed to PGE_2_ [68].

The presence of rosiglitazone in the prediction network, a thiazolidinedione drug used as an insulin sensitiser, might induce to hypothesise a possible direct or indirect involvement of bimatoprost with the peroxisome proliferator-activated receptors PPARs in mitochondrial membrane depolarisation. This hypothesis, which needs to be confirmed with further studies, appears to be consistent with the observation that patients undergoing anti-glaucoma therapy with prostaglandin analogues develop reversible periocular lipodystrophy [69,70,71]. In vivo studies in rats demonstrated that topical drops or retrobulbar injections of bimatoprost 0.03% induce atrophy of intraconal adipocytes compared with controls [72,73]. We can speculate that bimatoprost at high concentration (0.03%) may, directly or indirectly, negatively depress PPARs, plausibly PPARγ, inducing, as observed in mice knockout for PPARγ in adipose tissues, a lipodystrophic phenotype [74,75]. On the other side, bimatoprost at 0.01% seems to act positively on mitochondrial function.

Further and more in-depth studies are needed to clarify the mechanisms behind the hormetic effects of bimatoprost on conjunctival cells.

## 4. Materials and Methods

### 4.1. Cell Culture and Treatments

The Wong–Kilbourne derivative of the Chang human conjunctival epithelial (HCE) cell line (obtained from ATCC^®^, CCL 20.2; clone 1-5c-4; WKD; ChWK) was purchased by the European Collection of Authenticated Cell Cultures (ECACC 88021103). This cell line has been used previously for toxicological in vitro studies despite the presence of a small amount of HeLa cells, not affecting the cytotoxic response [76]. As previously described [50], HCE cells were routinely cultured at standard conditions (37 °C in a 5% CO_2_ humidified atmosphere), seeded at a density of 2 × 10^4^ cells/cm^2^, and maintained in Medium 199 supplemented with 2 mM glutamine and 10% heat-inactivated FBS (EuroClone, Pero, MI, Italy), penicillin 100 IU/mL, and streptomycin 100 µg/mL (Corning Inc., Somerville, MA, USA), until they reached a confluence close to 80%. Cells were maintained in standard conditions for 24 h before treatments. The cells were then exposed to BIM (Sigma-Aldrich, St. Louis, MO, USA) 0.3 mg/mL, corresponding to a final concentration of 0.03%, and BIM 0.1 mg/mL, corresponding to a final concentration of 0.01%, as detailed below. Stock solutions of BIM were prepared in phosphate-buffered saline-ethanol and stored in the dark at −20 °C. The negative control group was treated with vehicle control (vCTR).

### 4.2. Cell Growth and Viability

HCE cells were seeded at a density of 2 × 10^4^/cm^2^, and cultures were incubated at standard conditions in the presence or absence of BIM to reach a final concentration of 0.03% and 0.01% in the culture medium. Cell growth and viability were assessed at 24 and 48 h by Trypan blue exclusion assay (Sigma-Aldrich, St. Louis, MO, USA).

### 4.3. Flow Cytometry Analyses of Cell Cycle

Control (vCTR) and treated cells were collected and subsequently washed twice with ice-cold PBS, then fixed using a cooled 70% ethanol solution (Sigma-Aldrich, Saint Louis, MO, USA) in PBS, with gentle mixing at 4 °C for 30 min. Fixed cells (10^6^ cells/mL) were washed twice with ice-cold PBS and resuspended with a solution containing 50 µg/mL PI, 0.1% Nonidet-P40, and RNase A (6 µg/10^6^ cells) for 30 min in the dark at 4 °C. All reagents were from Sigma-Aldrich, Saint Louis, MO, USA. Data from 10,000 events per sample were collected and analysed using a FACS Calibur instrument (Becton Dickinson (BD) Instruments Inc., San José, CA, USA) equipped with cell cycle analysis software (Modfit LT for Mac V3.0) to calculate the percentages of cells in the G1, G2/M, and S phases.

### 4.4. Detection of Intracellular Reactive Oxygen Species (ROS)

The intracellular ROS production was detected by using 2’,7′-dichlorofluorescein diacetate (DCFH-DA) purchased from Molecular Probes (Eugene, OR, USA), as previously reported [51,77,78]. In brief, after 30 min and 2 h of treatments, samples were incubated with 1 µM DCFH-DA, at 37 °C for 30 min. Subsequently, cells were collected and twice washed in ice-cold PBS; samples were then analysed by flow cytometry to detect the presence of intracellular ROS, at the wavelengths of 502 and 524 nm, for the excitation and the emission, respectively. The fluorescence intensity was detected with a Perkin-Elmer LS-50B spectrofluorometer. Cells treated with 200 µM tert-butyl hydroperoxide (t-BHP) for 1.5 h were used as a positive control.

### 4.5. Assessment of ΔΨ_m_ by Flow Cytometry

Variations of the ΔΨ_m_ in samples exposed to BIM 0.03% and BIM 0.01% for 30 min and 2 h were assessed using the lipophilic cation dye JC-1, as previously described [52,79]. Cells (1 × 10^6^ per sample) were stained for 30 min at 37 °C in a humidified atmosphere with 3 µM JC-1 (Molecular Probes, Eugene, OR, USA) and collected; after washing in PBS, cells were analysed by flow cytometry. The cells were cultured in the presence of carbonyl-cyanide-3-chlorophenylhydrazone (CCCP) (Sigma-Aldrich, St. Louis, MO, USA) at a concentration of 8 µM for 1 h at standard conditions, and incubated with the potentiometric dye as described above, to ensure a positive control for the reduction of the ΔΨ_m_. Experiments were done in triplicate and analysed by a FACS Calibur instrument (Becton Dickinson (BD) Instruments Inc., San José, CA, USA). The fluorescent signals from JC-1 monomers or aggregates were detected through the FL-1 (525 ± 5 nm) and FL-2 bandpass filters (575 ± 5 nm), respectively; the forward and side scatter channels gated a minimum of 1 × 10^4^ cells on the major population of normal size-cells. ΔΨ_m_ data assessment was performed by the Cell Quest software (BD Instruments Inc.).

### 4.6. Annexin V/FITC and Propidium Iodide Assay

Control (vCTR) and treated cells (1 × 10^6^) were collected; after washing in ice-cold PBS, samples were resuspended in 1 mL of binding buffer (10 mM HEPES, 2.5 mM CaCl_2_, 140 mM NaCl, pH 7.4) containing 1 µg/mL PI and 10 µg/mL Annexin V-FITC. Cells treated with 25 µM Etoposide (Eto) for 24 h were used as a positive control for the induction of apoptosis. After staining (1 h incubation at room temperature), cells were washed and analysed by FACS Calibur instrument (Becton Dickinson Instruments Inc.). To calculate the percentages of apoptotic and necrotic cells, data from 10,000 events per sample were collected and analysed using a FACS Calibur instrument (BD Biosciences) equipped with cell cycle analysis software (Modfit LT for Mac V3.0).

### 4.7. Drug Network Prediction

The predicted functional partners from the drug network were determined by STITCH (Search Tool for Interactions of Chemicals database, http://stitch.embl.de/ (accessed on 11 November 2022)) using the input molecules reported in Table S2 in the supplementary file. Prostaglandin E_2_ (PGE2) and bimatoprost were added as input molecules. The malate-aspartate shuttle components (GOT2, MDH2, SLC1A3 and SLC25A11) and the mitochondrial uncoupling proteins (UCP1, UCP2 and UCP3) were added as input proteins. Results were filtered for *Homo sapiens* with a minimum required interaction score >0.7 (high confidence) and no more than 20 interactions. Text-mining, experiments, databases, co-expression, neighbourhood, gene fusion, co-occurrence, and predictions were included. Settings, more detailed information, and the interactive network plot can be retrieved at the following link: http://stitch.embl.de/cgi/network.pl?taskId=XymGQyDgGV6u (accessed on 11 November 2022). After the first round, rosiglitazone was added to input nodes to generate a second network with the same settings imposed in the first round. Settings, more detailed information, and the interactive network plot can be retrieved at the following link: http://stitch.embl.de/cgi/network.pl?taskId=ncdxT1FhAA9i (accessed on 11 November 2022).

### 4.8. Statistical Analyses

All analyses were performed in at least three independent experiments. Values reported in this study are expressed as the mean ± standard error (SE) unless otherwise indicated. The Sigma Stat 2.03 (SPSS, Chicago, IL, USA) was utilised to evaluate the statistical significance of differences between group means. All the comparisons between multiple groups were performed by ANOVA test (Dunnett’s method). A value of *p* < 0.05 was considered statistically significant.

## 5. Conclusions

In summary, our results do not indicate any cytotoxicity induced by bimatoprost 0.01% in terms of cell growth and viability, as well as mitochondrial activity, compared with a concentration of 0.03%. More investigations are required to understand better how in vitro data correlate to in vivo findings. However, considering both the causative role that mitochondrial dysfunction and oxidative stress have in the common ophthalmologic disorders affecting the ocular surface and retina and the extensive use of bimatoprost in anti-glaucoma pharmacotherapy, the use of preservative-free bimatoprost 0.01% in clinical practice may improve the tolerability and patient compliance to the drug therapy.

## Supplementary Materials

The following supporting information can be downloaded at: https://www.mdpi.com/article/10.3390/ijms232214113/s1.
