# Supplementary material for: Cytotoxicity, Mitochondrial Functionality, and Redox Status of Human Conjunctival Cells after Short and Chronic Exposure to Preservative-Free Bimatoprost 0.03% and 0.01%: An In Vitro Comparative Study
Source: Int J Mol Sci. 2022 Nov 15;23(22):14113. doi: 10.3390/ijms232214113 (PMC9695990; doi:10.3390/ijms232214113)
Supplement: Supplementary file 1 [file ijms-23-14113-s001.zip › ijms-2005049-supplementary.pdf]

Supplementary materials

Cytotoxicity, mitochondrial functionality, and redox status of human conjunctival cells after short and chronic exposure to preservative-free bimatoprost 0.03% and 0.01%: an *in vitro* comparative study.

Sabrina Petricca[1], Giuseppe Celenza[1,*], Ciro Costagliola[2], Fausto Tranfa[2], and Roberto Iorio[1]

1 Department of Biotechnological and Applied Clinical Sciences, University of L'Aquila, L'Aquila, Italy

2 Department of Neurosciences, Reproductive and Dentistry Sciences, University of Federico II, Naples, Italy

Correspondence* to Giuseppe Celenza, Department of Biotechnological and Applied Clinical Sciences, University of L'Aquila, Via Vetoio, 1, L'Aquila, 67100, Italy.

e-mail: **giuseppe.celenza@univaq.it**

Keywords: Bimatoprost; preservative-free; glaucoma; human conjunctival epithelial cells; cell cycle; mitochondrial activity; ROS; apoptosis

Table S1. List of the input molecules used for drug network prediction using the STITCH database. [a] PubChem CID for chemicals, UniProt code for proteins.

Molecule	Code[a]	Description
bimatoprost	127909	bimatoprost
PGE2	158	prostaglandin E2 (PGE2)
GOT2	P00505	glutamic-oxaloacetic transaminase 2, (mitochondrial aspartate aminotransferase 2)
MDH2	P40926	malate dehydrogenase 2, NAD (mitochondrial)
SLC1A3	P43003	solute carrier family 1 (glial high-affinity glutamate transporter), member 3
SLC25A11	Q02978	solute carrier family 25 (mitochondrial carrier; oxoglutarate carrier), member 11
UCP1	P25874	uncoupling protein 1 (mitochondrial, proton carrier)
UCP2	P55851	uncoupling protein 2 (mitochondrial, proton carrier)
UCP3	P55916	uncoupling protein 3 (mitochondrial, proton carrier)

Table S2. Predicted drug network functional partners from STITCH database (http://stitch.embl.de/) ranked by combined score, using the input molecules reported in Table S1. [a]PubChem CID for chemicals, UniProt code for proteins.

Node	Code[a]	Description	Neighbourhood	Gene Fusion	Co-occurrence	Co-expression	Experiments	Databases	Text-mining	Prediction	Homology	Combined score
**PTGER4**	P35408	*prostaglandin E receptor 4 (subtype EP4)*					•	•	•			**0.999**
**PTGER3**	P43115	*prostaglandin E receptor 3 (subtype EP3)*					•	•	•			**0.999**
**PTGER1**	P34995	*prostaglandin E receptor 1 (subtype EP1)*					•	•	•			**0.998**
**PTGER2**	P43116	*prostaglandin E receptor 2 (subtype EP2)*					•	•	•			**0.998**
**CS**	O75390	*citrate synthase*	•			•	•	•	•			**0.996**
**PTGS2**	P35354	*prostaglandin-endoperoxide synthase 2 (prostaglandin G/H synthase and cyclooxygenase)*						•	•			**0.995**
**FH**	P07954	*fumarate hydratase*	•			•	•	•	•			**0.994**
**oxaloacetate**	970	*oxaloacetic acid*					•	•	•			**0.993**
**glutamic acid**	611	*monopotassium glutamate (MPG)*					•	•	•			**0.992**
**pyridoxal phos.**	1051	*pyridoxal phosphate*					•	•	•			**0.990**
**malate**	525	*magnesium malate*					•	•	•			**0.990**
**carboxy**	283	*formate, (alias fatty acids)*					•	•	•			**0.989**
**phosphate**	1003	*Phosphoric acid*					•	•	•			**0.989**
**IL8**	P10145	*interleukin 8*						•	•			**0.988**
**MDH1**	P40925	*malate dehydrogenase 1*				•		•	•			**0.987**
**rosiglitazone**	77998	*rosiglitazone is an antidiabetic drug in the thiazolidinedione class of drugs*						•	•			**0.987**
**CXCR6**	O00574	*chemokine (C-X-C motif) receptor 6*					•	•	•			**0.986**
**LDHC**	P07864	*lactate dehydrogenase C*				•		•	•			**0.984**

**LDHB**	P07195	*lactate dehydrogenase B*				•		•	•			**0.984**
**ADCY2**	Q08462	*adenylate cyclase 2*						•	•			**0.983**

Table S3. Predicted drug network functional partners from STITCH database (http://stitch.embl.de/) ranked by combined score, including rosiglitazone as input molecule. [a]PubChem CID for chemicals, UniProt code for proteins.

Node	Code[a]	Description	Neighbourhood	Gene Fusion	Co-occurrence	Co-expression	Experiments	Databases	Text-mining	Prediction	Homology	Combined score
**PTGER4**	P35408	*prostaglandin E receptor 4 (subtype EP4)*					•	•	•			**0.999**
**PPARG**	P37231	*peroxisome proliferator-activated receptor gamma*					•	•	•			**0.999**
**PTGER3**	P43115	*prostaglandin E receptor 3 (subtype EP3)*					•	•	•			**0.999**
**PPARA**	Q07869	*peroxisome proliferator-activated receptor alpha*					•	•	•			**0.999**
**PTGER1**	P34995	*prostaglandin E receptor 1 (subtype EP1)*					•	•	•			**0.998**
**PTGER2**	P43116	*prostaglandin E receptor 2 (subtype EP2)*					•	•	•			**0.998**
**CS**	O75390	*citrate synthase*	•			•	•	•	•			**0.996**
**RETN**	Q9HD89	*resistin*						•	•			**0.996**
**PTGS2**	P35354	*prostaglandin-endoperoxide synthase 2 (prostaglandin G/H synthase and cyclooxygenase)*						•	•			**0.995**
**FH**	P07954	*fumarate hydratase*	•			•	•	•	•			**0.994**
**oxaloacetate**	970	*oxaloacetic acid*					•	•	•			**0.993**
**glutamic acid**	611	*monopotassium glutamate (MPG)*					•	•	•			**0.992**
**ADIPOQ**	Q15848	*adiponectin*						•	•			**0.991**
**malate**	525	*magnesium malate*					•	•	•			**0.990**
**pyridoxal phos.**	1051	*pyridoxal phosphate*					•	•	•			**0.990**
**carboxy**	283	*formate, (alias fatty acids)*					•	•	•			**0.989**
**phosphate**	1003	*phosphoric acid*					•	•	•			**0.989**
**LEP**	P41159	*leptin*						•	•			**0.989**
**CD36**	P16671	*platelet glycoprotein 4*						•	•			**0.988**
**IL8**	P10145	*interleukin 8*						•	•			**0.988**
